# Notices of the Principal Mineral Springs of Kentucky and Ohio

**Published:** 1828-06

**Authors:** 

**Affiliations:** Cincinnati


### Art. IV.—*Notices of the principal Mineral Springs of Kentucky and Ohio.* By the Editor.

The Mineral Springs of Kentucky and Ohio, especially of the former, are numerous and considerably diversified in their composition and qualities. They are but little known to the profession, even of those states; who, in general, are, therefore, unable to direct their patients to a proper choice. Having visited most of them, (though not with the means of an accurate analysis, nor for a sufficient length of time to observe all their effects) I propose, in this paper, to give a brief notice of those which constitute our principal *Watering Places.*

The countries on each side of the Ohio river, present an identity in the age of their rocks, but considerable variety in their composition. They are all secondary, and disposed in horizontal strata. The predominant rocks are grey lime-stone, abounding in organic remains; and sand-stone, alternated with shivers and slate-clay. The calcareous formation presents considerable variety in its geological character, as well as in its chemical composition. Springs are subterranean streams, supplied chiefly by the rains which percolate through the rocks over which they fall; and which, in their meanderings, dissolve such soluble matters as may be in their way; and hence the diversity, in quality, of the mineral waters, in the region of which I have spoken. Throughout almost every part of it, the common spring and well water, is *hard,*—in other words, is impregnated with the carbonate and sulphate of lime, the muriate of soda, and other saline substances. When any one or more of these, or other ingredients, are present, to a certain degree, the water is no longer regard as fit for culinary purposes; but pronounced medicinal, and resorted to by the valetudinary. In their selection, they are too often governed by considerations of convenience, or act in almost total ignorance of the relative effects of different springs. Thus they often suffer injury, when they expect to receive benefit. A mistaken choice in the watering place, is not, however, the only cause of disappointment. The conduct of the patient while there, and the exposures to which he is subjected, contribute greatly to an unfavorable result. Hence it is by no means uncommon, to see invalids who left home in hope, return in discouragement, if not despair. For the infirm to derive advantage from the use of mineral waters, several circumstances must be attended to.

1. It is necessary that the spring should be well chosen, in other words, adapted to the case of the patient.

2. That it should be in a healthy situation; or one, at least, as exempt as possible from the causes which induced the disease which is to be cured.

3. That the malady should be curable; or, if not curable, susceptible of palliation.

4. That it should not be of such a kind as to be aggravated by exercise.

5. That the patient should be comfortably lodged, in a dry and airy apartment, instead of being confined in a small cabin or room, close upon the ground floor, surrounded by high weeds and grass, in a damp valley, as is too often the case.

6. That he should not participate in the dissipation, which is so commonly practised by those who visit watering places for amusement only.

7. That he should be exceedingly temperate and simple in his diet. Mineral waters of almost every kind sharpen the appetite, sometimes to a morbid degree, and the majority of patients, suppose, or pretend to suppose, that while at a mineral spring, they may indulge themselves with impunity. Thus, in numerous instances, the beneficial effects of the water, are more than countervailed, by errors and excesses in diet.

8. That he should visit the watering place between the 15th or 20th of May, and the same period in September. The proper time, in this country, is the four months preceding the autumnal equinox; though, on the whole, it is better and safer to limit the residence to the three summer months. The first half of May is almost always too cool and rainy to be comfortable; and most watering places too much infested with fevers after the first of September, to justify a longer sojourn at them. These are given, however, as general rules, to which there are exceptions.

9. That the patient should remain longer than is usual. Of all men, the Anglo-American is, perhaps, the most impatient. He arrives at a watering place, and, like a thirsty animal, obedient only to his sensations, proceeds to drench himself. In three days he is, perhaps, worse than when he arrived; and, in a week, he decides, that the waters can do him no good, falls into displeasure with every thing about him, and repairs to another spring, or returns home in regret, at his useless expenditure of strength and money.

In most cases, mineral waters should be drunk moderately and perseveringly. Their specific effects, in many instances, are not perceived, nor, indeed, produced for some time. Their first impression, on many invalids, is unfavourable; but, by continuing them, the constitution, at length, becomes reconciled to their action, and is ultimately strengthened by it.

10. That in journeying to the watering place, he should proceed with deliberation, and not in a feeling of anxiety to reach the fountain upon which his imagination is turned, subject himself to long daily journeys and great fatigue. The end cannot justify such means. He should look to the travelling, not less than the water to which it is to conduct him, as a source of benefit; and, so perform it, as to increase instead of exhausting his strength.

11. That he should not place too exclusive a reliance on the water. It may be an important, without being the only, means of cure. In many cases, a particular course of diet is necessary, and should not be abandoned. In others, certain medicines are requisite, and should be persevered in.

By an attention to these, and such other rules as individual cases will suggest, mineral waters may do good in various cases; and, aided by the travelling, and the reviving and alterative influences of pleasant scenery and new social aspects, may, sometimes, effect that which all the drugs of the apothecary could not reach; and, what is still more, may avert the fate which, in chronic diseases, too often awaits him who is, from day to day, compelled to swallow them *en masse*. There are physicians who estimate the value of their interference, by the bulk and number of their boluses; and any spring, not absolutely impregnated with arsenic, would prove a fountain of health to all whom it might extricate from such keeping. These strictures, apparently severe, are directed against the practice in chronic diseases, only; but it is generally for them that mineral waters are prescribed.

Having premised these general remarks, on the use and abuse of mineral waters, I shall proceed to speak, in detail, of some of the principal watering places in the West.

HARRODSBURG SPRINGS.

The town of Harrodsburg, one of the oldest in Kentucky, is situated ten miles south of the river which bears that name, and near the geographical centre of the State. The site is elevated, rocky and rolling, but not hilly; and the surface of the surrounding country has the same character. Neither the town, nor its immediate vicinity, presents, in its scenery, any thing striking or picturesque; but within two or three hours ride, in different directions, the perambulating invalid may see several objects not unworthy of notice.

1. Union Village, inhabited by Shakers, who exhibit a characteristic specimen of the social, economical, and political relations of that singular people.

2. The spot denominated Knob-Lick, fifteen miles S. E. of Harrodsburg, five miles from the old and pleasant village of Danville, the site of Centre College; and two miles from the farm of the late venerable Gov. Shelby. The knobs or hillocks, are from one to two hundred feet high, more or less conical, some of them insulated, others connected by crumbling isthmuses,—the whole forming a group of barren, conoidal eminences, which are finely contrasted with the deep verdure of the surrounding plain. They consist of a marlaceous slate-clay, strongly inclined to disintegration, and reposing on shale.

3. The grey, mural cliffs of the Kentucky river, which flows in a narrow and winding ravine, nearly 400 feet in depth. This great natural canal, may be visited with facility, by several roads; and offers, in the grandeur of its high and precipitous banks, embellished with evergreens, a great deal, to interest all who have a taste for the sublime and beautiful.

But we must return to that which is more important to the invalid—

*The Springs*. These are four or five in number. They burst out near the summits of the ridges, on which the village of Harrodsburg, is built. The mass of these ridges is composed of lime-stone, much of which is of a fine grain, and impregnated with magnesia. All the springs afford water, essentially of the same composition; though the proprietors are accustomed to speak of them, as presenting some variety in their qualities and effects. None of the veins are large. They have no gaseous impregnation, and, consequently, do not sparkle. Their temperature is that of other springs, not more copious, in the same latitude—about 58° of Fahrenheit.

The water from one of them, on the land of Mr. Sutton, has been examined, with some care, by Dr. Best and myself. The following is a statement of the experiments made, and the results obtained; which, although not so rigorous and comprehensive, as to constitute a perfect analysis, will convey a correct general idea of the composition of all the fountains.

### EXPERIMENTS.

1. Prussiate of potash and infusion of galls produced a slight discolouration, both before and after boiling.

2. Nitric acid occasioned no change, nor effervesence.

3. Oxalic acid produced an obvious and diffused whiteness, which appeared slowly, and the same effect occurred after boiling.

4. Acetate of lead threw down a copious white precipitate, soluble in nitric acid.

5. Nitrate of silver produced the same kind of precipitate, soluble in the same acid, and, also, in ammonia.

6. The carbonate of ammonia caused a slight whiteness; and, upon the addition of the phosphate of soda, a copious white precipitate ensued.

7. One thousand grains of the water, evaporated, left a residuum of dry white matter, which, when apparently deprived of its water of crystallization, weighed twelve grains.

During the evaporation a slight pellicle formed on the surface.

8. A portion of this residuum afforded an effervescence with several acids, and was wholly soluble, in dilute muriatic acid.

9. Of this effervescent portion, nearly the whole was found to be soluble in sulphuric acid. The remainder was probably lime; which, united with the sulphuric acid, would remain undissolved. From this solution, the addition of carbonate of ammonia and phosphate of soda, threw down a copious white precipitate.

10. One thousand grains of the water were evaporated to one half. Lime water was then added, which produced a turbidness. The liquor was filtrated. Oxalic acid was then added to it, and occasioned a precipitate. It was filtrated again, and concentrated still further. Acetate of Barytes was then added, (not in excess) and produced a copious white precipitate. The solution was finally evaporated to dryness, and heat enough applied to decompose and drive off the acetic acid. The residuum, dissolved in water, changed the vegetable blues to green.

### CONCLUSIONS.

This water, it would seem, from the foregoing experiments, contains the following salts:

1. Sulphate magnesia, in large quantities. This is the characteristic ingredient.
2. Carbonate of magnesia, in a small quantity.
3. Sulphate of soda, do.
4. Sulphate of Lime, do.
5. Carbonate of Lime, in minute do.
6. Iron (probably in the state of a sulphate) a trace.
7. A minute quantity of sulphuretted hydrogen, as I ascertained by experiments, made at the spring itself.

From this analysis, it appears that the waters of the Harrodsburg Springs, bear some resemblance, in the materials which they hold in solution, to the celebrated Seidlitz fountain of Bohemia. Their predominant ingredient, is sulphate of magnesia, or Epsom salt; though the other matters which they contain, especially the sulphate of iron, small as it is in quantity, may contribute to their beneficial effects.

I am not in possession of the facts, necessary to a full *expose* of their therapeutick powers; but that these are so great as justly to place them at the head of all the known mineral springs, in the states bordering on the Ohio river, I have no doubt.

The cases to which they are, in a peculiar manner, adapted, are chronic inflammations, and obstructions in the abdominal viscera. Thus, they are, eminently serviceable, in such cases of dyspepsia, as are attended with subacute gastritis; in almost every kind of hepatic disorder, except when the liver is indurated, and, consequently, incurable; and in constipation, so constant an attendant on diseases of the stomach and liver. They are almost equally beneficial, in chronic inflammations of many other parts of the system—especially of the serous and fibrous membranes. In tonic dropsies, in rheumatism, and in various affections of the periosteum from febrile metastases, from syphilis and from mercury, they have often effected a cure, when other means had failed. In several urinary disorders they have done equal good. In chronic diseases of the skin, they have, also, been found useful, when the patient has been subjected to a regimen, that has determined them to the surface. In pulmonary complaints, they have been found serviceable; but not in the same degree, as in disorders of the abdominal organs; and their use in these maladies requires discrimination. In chronic pleurisy, and the early stages of subacute bronchitis, they have performed cures; but in vomica, tubercular suppurations, and hepatization of the pulmonary tissue, they are injurious; and, if persevered in, may even prove fatal. When they have rendered occasional assistance in these affections, it was chiefly by correcting a morbid condition of the digestive functions, so often associated with them. In sick headache they, occasionally, do good; but many cases of that obstinate malady, are attended with such an enervated condition of the nervous system, that their sedative operation becomes prejudicial. In headaches, dependent, however, on a phlogistic diathesis, they are salutary. Finally, in opthalmia, during the stages of acute and subacute inflammation—as long as depletives and sedatives are indicated--they may be relied upon as an efficient remedy.

It would be superfluous to cite other examples of their efficacy. The principle upon which they should be prescribed is obvious. Reducing the power of the heart, and acting as a diuretic, cathartic and sudorific, they are adapted to almost every case of inflammatory disease; while they are, contra-indicated, in most organic læsions, in exhausted states of the system from profuse discharges of every kind; and in all cases of broken up and ruined constitution. Of temperaments, the sanguineous and bilious bear them best: to the nervous and phlegmatic they are generally useless, not unfrequently offensive, and sometimes almost pernicious.

I have already indicated their *modus operandi.* Their influence on the circulation is, I believe, invariably that of a refrigorant and sedative. Their action on the kidneys is, in general, that of a powerful diuretic, in which the water itself, no doubt, performs an important, though not an exclusive, part. In the majority of persons, they, also, operate on the bowels, as a hydragogue cathartic; but those who are strongly inclined to constipation, or experience, in an uncommon degree, their diuretic influence, occasionally find it necessary to add to them a portion of the salts, which are prepared by evaporating the water, and are kept at the springs for that purpose. To determine them upon the skin, they should be taken, chiefly, at night; the patient lying in bed, with the surface of his body closely, but thinly covered. In these cases, two *small* matters require attention; for without an observance of them, the desired effect cannot always be obtained:

1. The forehead of the patient should be covered with a slip of flannel, as directed by Sydenham, when sweating is the object.

2. He should be left alone, or, at least, not be allowed to talk or read. If the waters be taken warm or hot, their sudorific effect will, in most cases, be greater, than if taken cold. Lastly, when all these means are unavailing, an opiate—

for example, the pulvis Doveri—should be employed, and will, in general, be successful.

It is a common remark, that these waters do not oppress the stomach. This is not owing to the presence of carbonic acid gas, or any other æriform fluid; but to their passing the pylorus, or being drunk up by the veins of the stomach, very soon after they are received into that organ. It is to the stimulus saline matters which they contain, that their early exit from it should be attributed.

It is proper to say a word or two, concerning the saline mass, which is obtained by the evaporation of these waters. It is called Epsom salt; but it, obviously, contains all the fixed ingredients of the springs, and might, if dissolved in a large proportion of cold water, form a medicated beverage, equally useful with that afforded by the fountains themselves. Indeed a dilute solution of these salts, in water impregnated with carbonic acid, and cooled with ice, would constitute a mineral water more palatable, and, perhaps, more efficacious, than those of Harrodsburg. Of these salts, considered as a substitute, for the refined and crystallized sulphate of magnesia, of of the shops, it would be right to make a most favourable estimate. I am by no means convinced, that any thing is gained on the score, either of taste or effect, by substituting the purified Epsom salt, for one rendered impure by the admixture of other saline substances; and I know, that many persons who have tried both, prefer the latter.

As this paper may fall into the hands of some, who do not belong to the medical profession, and as my object is utility, it may be proper to add, that the Harrodsburg waters are not antacid, in the true acceptation of that term. They are, popularly, said to contain magnesia; and are supposed to correct acidity of the stomach, in the same manner as the calcined or the lump magnesia. This, however, is a mistake. But a small proportion of their magnesia is united with carbonic acid, into a carbonate, which is a real antacid; the bulk of it is combined with sulphuric acid, forming Epsom salt, which cannot unite with the acid of the stomach; but may prevent its reproduction, by correcting the disordered condition of that organ, which causes it to be generated.

There are other Epsom springs in Kentucky, besides those of Harrodsburg; of which the only one that has acquired notoriety, is situated ten or twelve miles from that town, and one from Perryville. It is named the

### Rochester Spring.

It is a feeble but constant stream, that bursts out about 60 feet below the summit of a ridge of coarse grained, shell lime-stone. From the experiments and observations, which I have made upon this water, its sensible qualities, composition and effects, are so nearly the same, with those of the waters just described, that a detailed account of them would be superfluous.

### Olympian Springs.

In July, 1823, I spent a week at these springs, which constitute one of the oldest and most noted watering places of Kentucky; and what I shall say of them, is, chiefly, transcribed from notes made on the spot, at the time.

They are situated in Bath county, about 50 miles east of Lexington, on the waters of Licking river, which unites with the Ohio opposite Cincinnati.

On approaching them from the west, the country undergoes a change in its topography, geology and botany. The gentle slopes that give such beauty to the basin of Elkhorn, and the "country roundabout," of which Lexington is the metropolis, are succeeded by high and steep hills, the summits of which are narrow and serpentine. This is the commencement of the broken country; which, becoming more and more alpine, as we advance to the east, at last terminates in the Alleghany mountains. With this change of aspect, there is a corresponding change of structure. This first shows itself, on approaching Slate creek, between Mountsterling and the springs, where we observe strata and beds of arenaceous lime-stone, alternating with the blue, shell lime stone just passed over, and presenting organic remains of a different kind. The proportion of this new variety rapidly increases, as we advance; giving indication, at every step, of the transition which is at hand. This is to shale, slate-clay and sandstone; all of which are soon visible *in situ*. By the time we reach the springs, they become the predominant rocks, and the limestone which, just before, was seen imbedding them, now becomes the imbedded mineral. With the blue, shell lime-stone, the *arbustum terræ fertilis* disappears, and is replaced by the chesnut, quercitron oak, mountain chesnut oak, persimmon, pine, red cedar, and other trees, and shrubs not found in rich and marly soils, with a corresponding change in herbaceous vegetation. Thus the observing invalid finds himself transported, in a single day, into the midst of objects and aspects which are both new and curious.

As the study of nature, with the eye of poetry or of philosophy, may alleviate many of the ills, either real or imaginary, (if, indeed, the latter can have an existence) which impel us to visit watering places, it will not, I trust, he regarded as unprofessional, to furnish distant invalids with a rapid sketch of what will be presented to their observation in this quarter.

The valley in which the springs are situated, is expanded, at the place where they arise, to the breadth of half a mile; and depressed about 300 feet below the surrounding hills. A branch of Beaver creek meanders through it, and contributes to sustain the luxuriant and succulent vegetation, which overspreads all the alluvial grounds of the West. The view of this spot, on approaching it, is pleasant and picturesque. The surrounding country is broken; but the traveller has not yet passed over the most rugged part. This lies before him, and stretches forward to the east, without any visible limits. Immediately beyond the valley, he observes the hills to rise in greater elevation; and, destitute, in their precipices, of arenaceous lime-stone, to exhibit little else than shale and slate-clay, the latter of which, within an hour's ride, graduates into stand-stone. Two miles from the springs, in the direction of which I have spoken, is a detached and somewhat conical summit, that has received the name of Olympus. It may be regarded as a specimen of the region adjoining, and still further east. The road to Beaver creek Iron works, passes near the base of Olympus; and, at length, ascending from the valley, the observer finds himself at an elevation of of 6 or 800 feet; and sees around him nothing but deep and angular vallies, mural precipices, and rocky summits; which resemble ruined fortresses and towers. The ascent to many of these, is impracticable; but others may be scaled, and will reward the enterprise with views, which, to the inhabitants of a flat country, appear of no ordinary sublimity; while the coolness and freshness of the passing breezes, cannot fail to afford him relief from the enervating influences of a mid-summer sun. From one of these observatories I saw, in a neighboring valley, a rainbow, every part of which was beneath my feet. On the whole, however, the visitor should not expect many alpine phenomena—though his feelings will be pleasantly revolutionized by the wildness, novelty, and grandeur of the surrounding scenery.

The most interesting and valuable mineral of this region, is argillaceous iron ore, which exists in extensive beds, near the surface, and is smelted, at two or three places, on Slate and Beaver creeks.

A district of country so rugged and unproductive, has of course a thin population; and game is consequently not very scarce. The valetudinarian, therefore, who delights in hunting, has an opportunity to engage in it, with a good prospect of success; but without followiug him over the crags and fallen pines, which he must encounter, let us come to an examination of *the waters*, which have chiefly attracted him thither. These are supplied by several springs and wells; which present such differences in their composition, that of *all* the watering places of the west, this is supposed to afford the greatest variety.

I could not myself detect more than three kinds—a salt and sulphur, a white sulphur, and a chalybeate.

1. The salt and sulphur water is pumped up from a shallow well, near the margin of the brook. The temperature of the water as it issues from the pump is 58° of Fahrenheit. Its taste is that of a weak brine, moderately charged with sulphuretted hydrogen. Compared with the greater number of salines in the western country, the quantity of common salt which it contains is small, and the sulphuretted hydrogen is too little to escape from the surface in bubbles. When the neighbouring stream is swollen, its waters find their way into the well, which then affords a more dilute solution. It was in this state when my observations were made.

Subjected to the action of a variety of reagents, it afforded the following results; which, however, I do not state with confidence, in their accuracy.

1. Sulphuretted hydrogen.
2. Muriate of Soda, or common salt, and perhaps a little muriate of lime.
3. Carbonate of Soda.

I could detect no sulphuric acid, and, consequently, it contains no Epsom salts; and if either lime or magnesia be present, the quantity is exceedingly small.

2. The white sulphur spring, is situated half a mile from the well. It bursts out from a bank of shale, a short way up the side of a hill. Mr. Bankes, of Mountsterling, assured me that this spring made its first appearance during the earthquakes of 1811. Its temperature is 59°. Its composition is substantially the same with that of the well just described, but the ingredients of the two vary in their proportions. In the sulphur spring, the quantity of that substance is so great as to be deposited in the form of a whitish sediment upon the leaves and twigs which the water flows over; silver is more speedily tarnished than in the well; and the proportion of sulphuretted hydrogen, is sufficient to rise in bubbles to the surface; but still it is much less than in many other springs in Kentucky. On the other hand, the spring has but a weak impregnation of muriate of soda, compared with the well. The proportion of carbonate of soda seemed to be nearly the same in both. I could not detect in it, either sulphuric acid, or magnesia. The existence of iodine in sulphuretted waters, not having at the time of my visit, been made known, I did not of course, examine for them.

3. The chalybeate springs. These are two in number; and are situated about 40 yards apart; and half a mile from the salt and sulphur well. They burst out from between strata of arenaceous limestone, near the bank of a stream which a mile below mingles its waters with those of the brook already described. Their temperature, as the water issues from the rock in lively currents, is 52° of Farenheit. They deposit a reddish sediment. From a variety of experiments, on the water of the lower of these springs, I was satisfied that it contains nothing, but the carbonate of iron, with the proportion of muriates and carbonates, which our common springs afford. I observed the bottles, in which it was carried to the lodges of invalids, to be incrusted with the red oxide of iron, from the decomposition of the carbonate of that metal, and the escape of the carbonic acid, by the agitation. Thus many who thought themselves in the use of an efficient chalybeate, were drinking a water, which contained little else, than what exists in ordinary springs.

Besides the fountains, which I have described, there is another—a feeble vien—near the principal well, and called the *Vitriol spring*. It seemed to contain muriates and carbonates only, and these in such moderate quantities, that it is used for culinary purposes, although spoken of as medicinal. I could not discover in it, either sulphur, magnesia or sulphuric acid.

As I shall have immediate occasion to speak of saline-sulphuretted waters, more potent than those of the Olympian valley, and of a chalybeate spring more noted, than those I have just described, I shall say but little in this place of their curative powers. The following are the principal observations which I made on the spot.

The salt and sulphur water was chiefly drunk. From 1 to 3 tumblers full, were taken in the morning. It lay light on the stomach. Its diuretic effect was prompt and certain. Its action on the bowels was in most cases so inconsiderable, that common salt was added to increase its aperient qualities; and many persons found it necessary, besides, to dissolve in it a portion of Epsom salt. To some dyspeptic stomachs it was oppressive, even, in small quantities; and in one delicate female I saw it produce a tendency to syncope. In many persons it produced abdominal distension; and a few thought it the cause of more obstinate constipation, which arose perhaps from its great determination to the kidneys.

During my week's residence at the springs, I saw no invalids in a rapid state of recovery; nor heard any speak with applause of the beneficial effects of the water upon them. It is certain, however, that many interesting cures have been effected by it; although less powerful than some other springs of the same kind.

The water of the sulphur spring has been but little used, and its effects have not, therefore, been ascertained. They cannot differ materially from those of the water from the salt well.

The chalybeate spring seemed to be an object of secondary interest, and the resort to it was comparatively limited. Its effects, of course, are those which belong to all springs, impregnated with the carbonate of iron.

In summing up the particular advantages of a journey to this watering place, I would say, that they resolve themselves into the variety of waters in which it abounds, and into the novel objects and romantic scenery which it presents.

### Blue Licks.

At this watering place there are several fountains, all, however, of one kind, the salino-sulphurous. They are found on either bank of Licking river, 24 miles from the Ohio, and at the intersection of the former, by the high road, that leads from Maysville to Lexington. From the first settlement of the state till within the last 15 years, salt was manufactured at this place. The manufacture was rendered unprofitable by the increasing scarcity of fuel, and by the discovery of stronger water in western Virginia and Pennsylvania.

In many respects, the region about the Blue Licks, is one of the most remarkable in Kentucky. Its destitution of soil is such, as make it quite a phenomenon, to all who have been reared in the fertile parts of the state. For miles in almost every direction, rocks overspread, with mosses and lichens, cover the surface, which sends up little else, than stunted oaks, red cedars, briers, and sumach bushes. These rocks belong to the formation of blue shell limestone, which prevails so extensively in Kentucky,—they are, indeed, its upper strata, broken into fragments and left in place. That they once had a covering of loam and soil, like the other parts of the state, cannot be doubted, inasmuch, as we find spots upon which it is still retained. As my object is to depict the aspects of nature, instead of developing the causes which produce them, I shall bestow but a passing remark on those which have presented us with this surface of barren rocks, in the midst of such prevailing fertility.

The first adventurers to Kentucky, found the Blue Licks, the resort of almost every species of quadruped which dwelt in its forests. The herbivorous came to drink the waters and lick the soil, coloured blue by the sediment which they deposited; and the carnivorous, frequented the same place, to prey upon them. The number of deer, elk, and bison, was almost incredible. The last, especially, came in vast herds from distant parts of the country, forming roads, some parts of which are still visible. This, therefore, was a watering place from time immemorial; and judging from the osseous remains which have been dug up, the most ancient visitors of the spot, were the mastodon and arctic elephant. At certain seasons of the year, the bison, from a distance, were in the habit of sojourning at this place for weeks; and subsisting on the shrubs and herbaceous plants of the surrounding hills. To this destruction of the vegetables, which in all rainy and broken countries, are necessary to the stability of the soil, and to its being trodden up when wet, we may safely attribute, its descent into the vallies, and the consequent exposure of the rocky strata underneath.

Since the manufacture of salt has been discontinued, and fuel is no longer required, many parts of this little region, from which the trees had been cut down, have shot up dense groves of red cedar and sumach, which, in the absence of inhabitants, give to them the aspect of an infested wilderness. Such is a sketch of the scenery and ancient history of the Blue Licks. The invalid of romantic sensibility cannot but find at them, when, personally contemplated, not a little to interest him; while those who are curious in their inquiries into the predatory wars, which the first settlers of Kentucky were compelled to maintain, with the Indians, will have an opportunity, within two miles of their lodgings, to view the spot on which a most bloody battle was once fought. I will only add, that to those who are fond of fishing, the river affords opportunities for angling, which are not to be enjoyed at any other public watering place of the state.

*The Springs* themselves, are more copious than the Olympian, just described; the water is much salter, and sparkles briskly, with sulphuretted hydrogen gas. Much of it is taken, in barrels, to the surrounding towns, even to Lexington—a distance of 40 miles—where it is held in good estimation. When kept in this manner, however, the dissolved sulphuretted hydrogen escapes, and the sulphur, remaining suspended or deposited on the sides of the vessel, gives to the water a more offensive taste, than it has at the springs, while its medicinal virtues, are no doubt diminished. Of these I shall not speak, till I have given some account of another watering place of the same kind; and the last which I shall describe in Kentucky. It is

### Big Bone Lick.

This spot has acquired a notoriety that is not even limited to the United States. Its name explains the nature of this distinction. No place in America, perhaps none in the world, has afforded an equal number of large fossil bones, of which the most conspicuous, belonged to the mastodon and the northern elephant, though the whole are included, by the people, under the designation of Mammoth bones. They were enveloped in the mud of a narrow valley, through which the salt and sulphur water rises in a number of streams, some of which are copious. Through this valley, a stream descends from the surrounding hills, and mingles with the Ohio, at the distance of 2 or 3 miles. When the river is swollen, however, the 'backwater' is found quite at the springs. They lie in Boone county, 22 miles from Cincinnati by land, and twice that distance by water. The surrounding hills are romantic, but there is no distant perspective, and although the scenery is superior to that of Harrodsburgh, it is inferior to the Blue Licks or Olympian springs. The deepest interest which the spot can inspire, arises from the osseous relicks, which its alluvial deposites afford.*

*The Waters* of this valley were once evaporated for salt, but the manufacture has been discontinued. They afford a larger quantity of muriate of soda or common salt than those of the Olympian springs, and abound in sulphuretted hydrogen gas, which is incombustible. They contain muriate of lime which also exists in the Blue Lick water and probably in the Olympian; and which, when salt was manufactured at those places, constituted a *bittern* that proved poisonous to cattle. They, likewise, contain a sulphate; but whether of soda or magnesia, I had not the means, when there, of ascertaining. They have not been tested for iodine. In a notice of the waters of Big Bone which I inserted in the picture of Cincinnati, in 1815, it is erroneously stated, that they contain gallic acid. The colouring of certain metallic precipitates, which I attributed to that acid, was no doubt owing to the sulphuretted hydrogen. They contain no iron.

* The first huge fossil bones ever taken from America to Europe, (unless some of those described by Dr. Cotton Mather, the early historian of New-England, as the bones of antideluvian giants, might have been carried from the colonies to the mother country) were found at this place; and some of them fell into the hands of Dr. William Hunter. This was about the middle of the last century. Since that time the spot has been explored, like another Herculaneum, and its disinterred relicks, have been disseminated over more countries, than the lost animals, perhaps, ever inhabited. The principal collections have been disposed of in the following manner. 1. That made by my precepter Dr. Goforth, in 1803 was, credulously, confided, in 1806, to the infamous, English traveller Thomas Arville, alias, Ashe, to be exhibited, in Europe. On his arrival at Liverpool, he sold on his own account, the entire collection, to Mr. William Bullock, who soon afterwards transferred his Museum to London. A part of them, were exchanged, by him, for other subjects of natural history, with the curators of the Royal College of Surgeons; a few were presented to Dr Blake of Dublin, and to Prof. Monro of Edinburgh; and the remainder were sold, with his other curiosities, at auction. 2. The collection made by order of Mr. Jefferson, as President of the American Philosophical Society, about the year 1805, was divided between the society, and M. Cuvier, the Naturalist of France; who received the most interesting portion of them. 3. The acquisitions made, by the Western Museum Society in 1819, are now in the Western Museum. New explorations would, no doubt, show that many bones yet remain, as Mr. Letton has, within the last two years, added a number, to the Cincinnati Museum.

It is not unworthy of remark, that Mr. Bullock, is now a landed proprietor and resident, of the same county, in which Big Bone is situated.

*The Modus Operandi* of the Big Bone and Blue Lick waters is identical, and not materially different from those of the Olympian springs, and of Harrodsburg They are more energetic in their action than the former, and more pervading in their effects than the latter. Thus, it is not difficult to direct them to the skin; and it frequently happens, after their use for a few days, that an eruption takes place, and boils, of different sizes, form in the subcutaneous cellular tissue. These effects are especially experienced by those who bathe in them, hot. Like the Harrodsburg waters, they lower the pulse, augment the urinary secretion, and produce copious liquid alvine dejections. To obtain the last effect, however, many persons are compelled, in the early stages of their residence at the springs, to resort to magnesia or Epsom salt. They increase the appetite, and do not oppress the stomach by their weight, except when that organ is greatly debilitated; but they frequently excite gastric acidity, in the dyspeptic. It would seem that they should increase the secretion of bile, but I have not been able to satisfy myself that this is the fact during their use: the great dilution to which the excreta are subjected, may, however, have led to this uncertainty. I have not often seen invalids much benefitted by them, until after their return home. A period of better health, then, in general, succeeds, provided, the remedy was adapted to the disorder. In a case of subacute trachitis, I saw them effect a cure; but in true pulmonary consumption, and in scrophula, except in the early stages, they are prejudicial; and might prove fatal, if used at a late period. In chronic affections of the liver, in cutaneous disorders, and, in many cases of amenorrhœa, they are signally serviceable. In chronic rheumatism, they have often done good. Where relief from a costive habit is the object, they should be used for several weeks, when they will often prove effectual. To enumerate all the cases to which I suppose them to be adapted, and in which they should be forbidden, would require ampler opportunities for observation than I have possessed; and in a paper addressed, chiefly, to the profession, would, perhaps, be unnecessary. I shall, therefore, dismiss the subject of their medicinal effects, and close my notice of the watering places of Kentucky, by a few remarks on their relative salubrity and comforts.

Kentucky, like the other States of the Union, has its summer choleras and autumnal fevers; and, although these, in common years, prevail every where, it is undeniable that some parts are more affected than others; especially with the latter class of diseases. Now, when the waters of the five situations which I have described, are so nearly alike in their effects, that the invalid might choose with reference, chiefly, to the healthiness of the place where he must sojourn,—which place should he select? As a witness in the interests of patients, and not of proprietors, I have no hesitation in testifying in favor of Harrodsburg and Rochester, over the Olympian Valley, Blue Licks, or Big Bone. The invalid resides, at either of the first, on dry and elevated ridges; at all the others, in deep and humid valley. Bilious fevers may occur, with fatal malignancy, at the former; but they are still more likely to happen in the latter, while ague and fever, which so seldom prevails on the ridges, almost annually infests the valleys, from the beginning of September, till white frosts have set in.

Further. In the facilities which they afford for taking carriage exercise, Harrodsburg and Rochester have an equal advantage over the other watering places of the state; and a like superiority might be awarded to them, especially to the former, in reference to the society afforded by the surrounding country. As to the society at the springs themselves, it will in general be nearly the same, throughout the whole. Creating itself, *de novo*, in every succeeding summer, it must of necessity be according to the new elements, which compose it; and over these no individual can exercise either physical controul, or moral supervision. It has, however, an adjunct which the proprietors might prune away; and it is to be regretted that they do not emulate each other, in the work of reform. I refer to the gaming machines, and gambling banditti, who periodically infest these places. They call off the attention of husbands, fathers, and brothers, from those whom they had conducted thither for health; they draw the unwary into their snares with the greater facility, because of the idleness which prevails at such places; in fine, the very rumour of their presence, is offensive to the taste and feelings, of moral and religious invalids; and has often banished them from the springs, before a proper trial was completed. Let it not be said that, in these strictures, I am travelling beyond the limits of a professional paper. Medicine is no longer the art of astrology or coscinomancy; but a liberal science—comprehensive, searching, exhortatory: embracing in its periscope the entire moral and physical man: flourishing most in the bosom of the highest civilization: casting its glances into all the natural sciences, and useful arts—upon social institutions, morals and religion: correcting abuses, rectifying errors, exposing absurdities: discovering every where the semina of diseases, and pressing into its service, the circumstances of the moral world, not less than the productions of the natural. The fabulous Argus, with his hundred eyes, would not be an inappropriate emblem, for a profession which must explore all the labyrinths of nature, and contemplate every aspect of society.

Before taking leave of Kentucky, I ought to add, that among its springs, not yet extensively known, is one only four miles from Cincinnati, which is said to be of the same composition with those of Harrodsburg. It is situated in a healthy spot, and has, already, though of recent discovery, attracted considerable attention. In an early number of this Journal, I shall publish a further notice of it.

The geography of Ohio, not less than its institutions, differs, in several points, from that of Kentucky. With the exception of its S. E. portion, and its southern border contiguous to the river, it is much leveller than Kentucky. Its valleys are generally of great width; and it has extensive plains, either horizontal, or slightly inclined to the north or south, according as the streams which traverse them, tend to Lake Erie, or to the Ohio river. In its geology the differences are, also, considerable. The hilly portions east of the Scioto river, in the southern part of the State, abound in shale and sand-stone, with salt, iron and coal; being, in fact, a part of the same formation in which the Olympian springs, of Kentucky, are situated. The other parts rest on blue shell, and arenaceous lime-stone, like the northern portions of the sister State; but the former has its laminæ separated by blue, stone, marl, which, in many places, becomes the predominant rock; and, greatly disposed to crumble down, has offered to the waters an easy conquest; and hence, in part, the extraordinary width of our valleys. It is, however, near the surface, that the greatest dissimilarities in the geology of the two States are to be found. Kentucky has, nearly every where, a thin covering of loam; its streams generally flow in narrow valleys, and it is, almost, without alluvial deposites—at least of sand and foreign, rolled pebbles. In Ohio, on the other hand, the rocky, calcareous strata are, in general, deeply buried up in loam; and in its expanded valleys, the quantity of water-worn rocky *debris*, both indigenous and exotic—to borrow the terms of a kindred science—is almost incredible.

The mineral springs of Ohio are numerous, though not greatly diversified in their properties, nor copious in the supplies which they afford. But few of them have yet attracted the attention of the profession, or been made places of public resort. In the shale and sand-stone region, there are salines; but I am not aware that they are frequented by any great number of invalids. The most common mineral waters of the State, are chalybeate. Feeble veins of this kind are every where met with, along the banks of our alluvial streams; and are, evidently, formed on the spot, by the percolation of rain water, through deposites abounding in vegetable recrements, and supplying oxide of iron and carbonic acid. Our arenaceous lime-stone, affords a water of the same composition, in large and perennial streams. Of these, one only, has, in any degree, fixed the attention of the public; and to it, as the only watering place of notoriety, in Ohio, I shall confine my remarks.

THE YELLOW SPRING

Is situated in Green county, 64 miles north of Cincinnati, and 2 miles west of the Little Miami river. It is a copious and constant fountain, that issues from between strata of arenaceous lime-stone, and thus has geological characters, perfectly identical with the chalybeate springs, of the Olympian valley, in Kentucky. It bursts out near the summit level of that quarter, but is soon precipitated into a wild ravine, more than a hundred feet in depth. On its way, thither, it has, in the course of ages, deposited a great bank, of reddish yellow ochre, which is intermixed with twigs and leaves of trees. The temperature of this fountain is 52°—precisely that of other springs in the neighbourhood, which afford a great supply of water, and issue from crevices in the rocks. The water is transparent, does not sparkle, and has a slight chalybeate flavour; but it gives very little offence to the taste, and is used for a variety of domestic purposes. Its component parts are those of all the springs of a lime-stone country, with the addition of proto-carbonate of iron; or the black oxide of that metal, dissolved by the agency of carbonic acid; which, however, is not in such quantity as to be emitted in bubbles. After the water has left the aperture from which it issues, a portion of the gas combined with it escapes, and a part of the oxide of iron, passing from the first to the second degree of oxidation, is deposited,—constituting the ochreous sediment, of which I have spoken. When the waters are transported to a distance, the same thing occurs; and if they be carried far and much agitated, the oxide of iron may be so entirely thrown down, that the most delicate tests will scarcely indicate in the water, even a trace of that metal.

Of all the sensible effects of this water, its diuretic are the greatest; but whether, in reality, they are much more considerable, than those of common water taken in the same quantity, may be doubted. As a laxative, its powers are very small. It has no necessary determination to the surface of the body. Taken even in large quantities, it does not oppress or embarrass the stomach; but improves the health of that organ, sharpens the appetite, augments the powers of digestion, and increases the general strength. To obtain from it, however, these salutary effects, it is necessary, by means of Epsom salt or magnesia, to maintain a regular habit of body. By adding the former to it, in the proportion of a tea-spoonful to the quart, a valuable laxative and tonic medicine is obtained.

The cases, to which this spring is adapted, are not those which require the waters of Harrodsburgh or Big Bone. It will not reduce inflammation; and, if there be unsubdued and smothered fires lurking in any of the organs of the system, they must be extinguished, by a saline water, or some other means, before the invalid ventures to the Yellow Spring. Its waters are, indeed, rather restorative than curative. They are admirable for convalescents. They belong to the class of tonics; and if not the most potent, are among the most pleasant. In many cases of dyspepsia, morbid sensibility of the stomach and bowels, torpor of the liver, atonic dropsy, hysteria, hypochondriasm, and other nervous affections; in the exhaustion from profuse evacuations; in the debility consequent upon fevers; and, finally, in most cases of enervated and broken up constitutions, they are likely to produce effects the most beneficial.

As a place of residence for the *three summer months*, the Yellow Spring has much to recommend it. Being the most northern watering place of the west; and, elevated more than 800 feet above the level of New-Orleans, it offers as cool a retreat for itinerant valetudinarians, as can be had short of Saratoga; while from its topographical circumstances, it should be a healthy situation. After the first of September, however, its nights become uncomfortably cool and damp; when visiters from the south, may as a general rule, prepare to leave it. My own opinion is that in September and October they are more likely to enjoy good health in the city, than out of it.

The country, about the spring, is so level, as to permit the invalid to take exercise in various directions; and the villages of Xenia, Springfield, and Dayton are all within his reach. He may visit the two first and return on the same day. Of natural scenery, he will be presented.—First with the valley, into which the spring discharges its waters. The brook that flows in this wild and narrow channel, falls over many successive ledges, constituting a series of little cascades; while its margin is fringed, (or formerly was) with a variety of beautiful shrubs, whose broad and heavy foliage, affords an agreeable contrast with the slender leaved cedars that adorn the cliffs of arenaceous limestone rocks above. Secondly, with prairies, many of which exist not far north of the spring, and display in the splendor and variety of their summer flora, enough to delight all, who have a taste for the contemplation of nature's fine arts. Thirdly, with the falls of the Little Miami, which are two miles from the spring. They will be found an interesting curiosity. The water falls in the course of a mile or two, not less than 200 feet.

"The stream is seen entering a chasm in the arenaceous limestone rocks, which underlay that quarter; in the course of a mile it is precipitated from several successive tables, when, being compressed to less than ten yards in width, it falls from a ledge of rocks, 6 or 8 feet, into a narrower fissure, of such great depth, that for several rods below there is no perceptible current. The sides of this fissure, which rise by estimation 50 feet above the surface of the water, are irregular, but correspond in such a manner as to suggest that they were formerly in contact. From this point, the rapids continue more than a mile. The chasm, widening and deepening, gradually terminates in the broad valley through which the stream afterwards flows in the Ohio. These effects seem to have resulted from the action of the current below, and the expansion of freezing water in the fissures above, which, operating in conjunction, have covered the steep acclivities with enormous masses of rock, whose former situations are still visible. These fragments and the superincumbent cliffs are decorated with four different evergreens—the Red Cedar, Canadian Yew, Hemlock and American Arbor Vitæ, interspersed with several other common trees and shrubs, which give to the scenery an aspect equally beautiful and romantic."—*Picture of Cincinnati—p.* 35.

It has not been my design to speak of the accommodations provided for invalids, at the different watering places of the west; but as they have heretofore been exceedingly limited at this spring, it may be proper to say, that they have recently been made every way correspondent to the natural comforts and advantages which are offered by that place.

With these remarks I close, a paper, written in too much haste to be made concise; and perhaps abounding too much in references to scenery and geological appearances to meet the approbation of the *severe* cultivators of our science. I cannot in conscience offer it, as a model, to my brethren of the west; but hope it may stimulate some of them, to deeper researches and happier efforts on the same subject.

*Cincinnati, June* 24, 1828.

